# MRI-based cortical morphometry in traumatic brain injury: a pilot longitudinal study

**DOI:** 10.1007/s00234-026-04140-3

**Published:** 2026-08-03

**Authors:** Matthew Boulton, Ali Al-Rubaie

**Affiliations:** 1 School of Health Sciences, Swinburne University of Technology, Melbourne, Australia; 2 Department of Medical Imaging and Radiation Sciences, School of Primary and Allied Health Care, Faculty of Medicine, Nursing and Allied Health, Monash University, Melbourne, Australia

**Keywords:** TBI, FreeSurfer, cortical thickness

## Abstract

**Purpose:**

Traumatic brain injury (TBI) can result in various changes to cortical morphometry which may negatively affect quality of life for individuals with TBI. Previous research has identified both increases and decreases in cortical volume, surface area and thickness across brain regions, suggesting that the impact of TBI on cortical morphometry remains unclear. This project used the “recon-all-clinical” pipeline on 4 mm Axial MRI scans to investigate cortical volume, surface area and thickness following TBI, which is important for improving understanding of post-injury neuroanatomical changes and refining neuroimaging methodologies.

**Methods:**

The measured regions were grouped using area-weighted means into cortical lobes consisting of frontal, parietal, occipital, temporal, cingulate gyrus and insula for analysis. The changes in morphometry were analysed longitudinally in group with TBI and compared with age/sex matched control scans.

**Results:**

The findings showed significant cortical thinning over time in the group with TBI in the left occipital cortex and right cingulate gyrus, and no significant changes in cortical surface area or cortical volume across any of the measured regions following corrections for multiple comparisons. When comparing individuals with TBI and neurotypical control, this revealed increases in cortical thickness in the left insula in the initial TBI scan compared to neurotypical control.

**Conclusions:**

The increased thickness at initial TBI scan should be interpreted cautiously, as they may reflect segmentation uncertainty in the presence of lesion heterogeneity, altered tissue contrast, and imaging artefacts. Additionally, potential early post-injury neuroplastic or compensatory changes may contribute to these findings, rather than indicating true morphometric differences. After applying corrections, significant longitudinal reductions in the left occipital cortex and right cingulate gyrus were found, suggesting that these regions may be vulnerable following TBI. These results suggest that the complexity of cortical morphometry changes immediately following TBI may reduce the validity of these measurements. Future studies should investigate this further with a larger sample, to further determine the usefulness of “recon-all-clinical” for short-term morphometric analysis post-TBI.

**Supplementary Information:**

The online version contains supplementary material available at https://doi.org/10.1007/s00234-026-04140-3.

## Introduction

Changes in cortical morphometry can impact quality of life for patients following TBI, with studies finding that cortical thickness reductions in the left parietal, temporal and occipital areas post-TBI were associated with significantly impaired accuracy of facial memory [1]. Poor sleep quality has also been associated with decreases in cerebellar and cortical volume [2], and decreases in cortical thickness bilaterally in the frontal, cingulate and precuneus regions, and in the right temporal and parietal regions [3].

Following TBI the brain undergoes structural changes to adapt to the trauma; such as changes in cortical volume following mild TBI (mTBI), however brain morphometry may increase or decrease following TBI depending on the injury location and severity [4, 5]. Researchers have examined how cortical volume changes following mild and moderate-severe TBIs. Several studies have reported global volume reductions within 3 months post-TBI, with no significant differences with respect to the GCS of the patient [6], with others finding similar decreases in global cortical volume following mTBI [7]. Beyond global measures, regional cortical volume reductions have been consistently reported in the frontal, parietal and occipital lobes when compared to healthy controls [8]. These regional reductions have been associated with poorer retention of newly learned material, as well as poorer working memory and executive control [8]. Further reductions have been observed across various cortical regions, including portions of the cingulate gyrus, right insula, and bilateral frontal and temporal lobes [9]. More detailed analyses of cortical gyri and sulci have revealed volume reductions in the left insula, right lateral and medial orbitofrontal cortices, the precentral and postcentral gyri, the rostral anterior cingulate cortex, and the transverse temporal gyri in individuals with TBI relative to controls [10]. However, not all studies report evidence of cortical atrophy. Longitudinal investigations have found no significant cortical volume changes at 2-week and 4-month follow-ups following mTBI [11], highlighting inconsistencies across studies. While cortical volume provides valuable insight into structural changes following TBI, it may be further decomposed into cortical surface area and thickness to more precisely characterise the underlying morphometric alterations [12].

Studies analysing cortical surface area changes following TBI have yielded mixed and region-specific findings. Some investigations have reported increased cortical surface area in regions such as the right lateral occipital gyrus following mTBI [13], while others have identified significant surface area reductions in the orbitofrontal cortex alongside increases in the posterior cortical regions [12]. Similarly, the literature on cortical thickness changes following TBI demonstrates substantial variability, with studies reporting cortical thinning in the fronto-temporal regions following mTBI [4]. Longitudinal research has identified thickness reductions in areas such as the left rostral middle frontal, left caudal middle frontal, and left lateral orbitofrontal cortices in individuals with moderate cognitive impairment two years post-injury [14]. In contrast, some studies have found no significant cortical thickness changes following mTBI [13], suggesting that thickness alterations may not be universally presents, or may depend on injury characteristics and study methodology. Both acute and long-term effects have been documented, with one longitudinal study reporting significant cortical thinning in the left middle temporal and right superior parietal regions at baseline following mTBI, with reductions in the left middle temporal region persisting at follow-up [15]. In military populations with blast-related TBI, clusters of cortical thinning have been observed in the right insula and inferior temporal and frontal regions, with no significant differences observed between mild and moderate TBI severity groups [16]. Mazaharally et al. [12] investigated cortical morphometric changes in 67 individuals with clinically confirmed TBI across 4-time points ranging from 5 months to 7 years post-injury, compared with 18 controls whose scans were taken at 2-time points. Their findings revealed increased cortical thickness in the frontal regions, alongside cortical thinning in the posterior cingulate and precuneus regions [12]. Together, these findings suggest that the cortical morphometric responses to TBI are region-specific, may differ between surface area and thickness measures, and can evolve from the acute to long-term phases post-injury. Cortical thickness changes vary across studies, time since injury and injury severity. Therefore, current understanding of how cortical morphometry may change following TBI remains incomplete. Furthermore, TBI may alter the typical age-related patterns of cortical thinning and surface area reduction, indicating a complex relationship between injury-related processes and long-term cortical remodelling [5].

Many of the above studies have used FreeSurfer, and specifically the “recon-all” pipeline to analyse cortical morphometry following TBI [1, 5, 12, 17]; one main limitation in analysis of cortical morphometry is the requirement of high quality, research grade MRI scans with 1 × 1 × 1 mm thickness to run this analysis smoothly. However, new tools have become available on FreeSurfer published by Gopinath et al., named the “recon-all-clinical” pipeline, which allows for advanced cortical analysis on hospital grade MRI scans ranging from 1 mm to up to 6 mm slice thickness, however the accuracy degrades as the thickness increases [18]. This technology may substantially expand the applicability of analysis of brain structure following TBI, as it allows for the measurement of large sample hospital grade MRI scans which may range from 1 mm to 6 mm in slice thickness, however at this stage no papers have investigated cortical morphometry following TBI using the “recon-all-clinical” pipeline. Due to the use of 4 mm slice thickness for every scan in this project, rather than using the various sub-regions of the Desikan-Killiany atlas [19], the specific areas investigated in this study have been separated into cortical sub-regions consisting of the frontal, parietal, temporal and occipital, alongside the cingulate gyrus and insula, which is discussed further in the Methods Section.

This study aims to demonstrate the use of “recon-all-clinical” for individuals with TBI in a small-sample study to investigate how cortical surface area, cortical thickness and cortical volume of the lobar regions changes over time following TBI, while also comparing this with age/sex matched controls. It is hypothesized that following TBI, there will be longitudinal reductions in cortical surface area, volume and cortical thickness. It is further hypothesized that cortical surface area, volume and thickness will be reduced in individuals with TBI at both time-points when compared with age/sex matched controls.

## Methods

### Inclusion/exclusion criteria

The scans consisted of retrospective patient T1 MRI scans from the Alfred Health, a major publicly funded healthcare network in Melbourne, Australia, with the following criteria:

1. Diagnosed with a traumatic brain injury (defined as an alteration in brain function or other evidence of brain pathology caused by an external force).
2. Had a mild, moderate or severe level of brain injury.
3. Had at least one follow-up MRI scan.
4. Had an MRI without artifacts.
5. Had no pre-existing history of brain disorders which could impact brain volume measurements, including prior brain injury, neurological conditions, long-standing psychiatric conditions of concurrent substance or alcohol abuse.
6. Aged 18 and older.

The above criteria were selected based on prior literature which have used similar criteria to include/exclude TBI cases [15, 20, 21]. These criteria were decided in a pre-defined methodological design, which was provided to the researchers at the Alfred Health prior to the allocation of participants.

## Image acquisition/analysis

36 patient MRI scans were obtained from the Alfred Health with approved human ethics #684/24. Among these, 12 clinically diagnosed individuals with TBI were included, each with both baseline and follow-up imaging, resulting in 24 TBI scans. However, 1 case was excluded due to haematoma’s which complicated cortical reconstruction and segmentation, and two cases had their left or right hemisphere removed due to haematoma and/or severe structural changes which impeded accurate measurements. The 11 resulting individuals with TBI were examined using 1.5 Tesla MRI scans, which were obtained from routine checkups following TBI at two timepoints which varied depending on the participant. The first scan was immediately following TBI (0–21 days), then one follow-up scan was completed, which ranged between 4 months and 5 years depending on the patient history with an average of 20 months, however most cases were between 6 and 18 months, with a median of 12 months. Due to the retrospective nature of this study, follow-up scan timing was determined at the discretion of the clinicians.

The group with TBI consisted of 7 male and 4 female, with ages ranging from 19 to 60. Glasgow Coma Scores (GCS) ranged from 3 to 14; Mild TBI was categorised by a GCS of 13–15 with a median GCS of 13 (*n* = 5). While Moderate-Severe TBI was categorised as GCS of 12 or below, with a median GCS of 3 (*n* = 6). A total of 12 control cases were allocated to compare with the TBI measurements with a single baseline scan, these cases were sex matched with 8 male and 4 female and were perfectly age-matched with the group with TBI.

All scans were stored in a secure Alfred OneDrive and were converted into Digital Imaging and Communications in Medicine (DICOM) data, and all cases had slice thicknesses of 4 mm for the axial T1 scans for consistency.

## Measured Brain Regions

Cortical reconstruction and morphometry segmentation was performed using FreeSurfer 8.0.0 (https://surfer.nmr.mgh.harvard.edu/). This project used a new processing tool named ‘recon-all-clinical’, which follows similar steps to ‘recon-all’ however is capable of measuring brain morphometry on scans up to 6 mm slice thickness [18, 22]. This pipeline undergoes various processes, including SynthSR which turns heterogeneous clinical MRI scans into T1WI for 3D morphometrical analysis [23], SynthSeg which allows for the segmentation of brain MRI scans of any contrast or resolution, without the need to retrain the model [24, 25]. Following this, the steps consist of the typical recon-all steps, including skull-stripping and the removal of non-brain tissue, intensity normalisation and segmentation, allowing for cortical reconstruction and measurement [18]. Following this process, amongst many others, a white matter surface map is created, alongside a pial surface map which can be used to visualise cortical morphometry.

## Cortical Morphometry Measurements

**Fig. 1:**
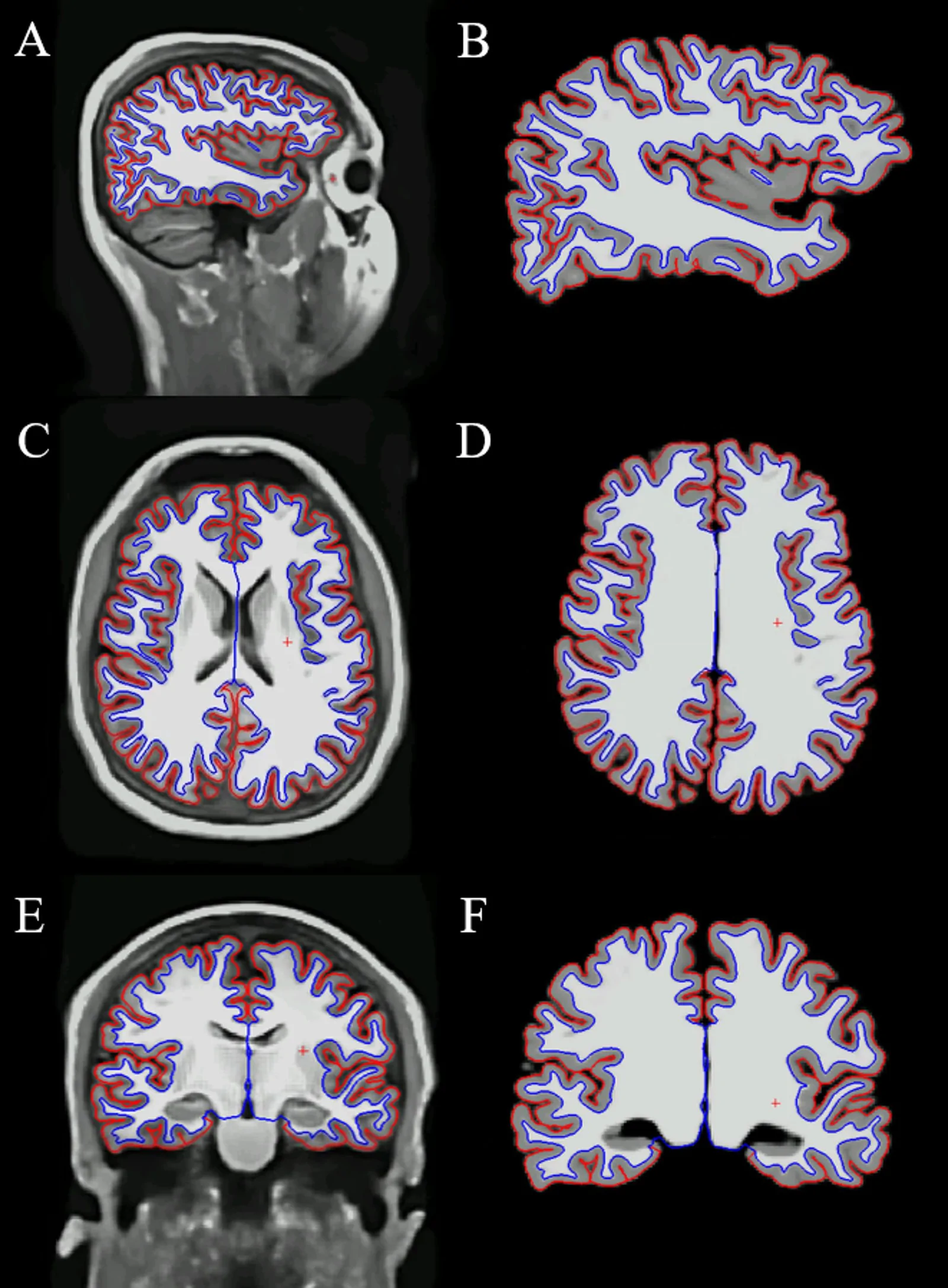
(A) Sagittal SynthSR Raw image used for quality control, showing pial and white matter surfaces, (B) Sagittal reconstruction of brainmask with grey and white matter segmentation, alongside pial and white matter surfaces, (C) Axial SynthSR Raw image used for quality control, showing pial and white matter surfaces, (D) Axial reconstruction of brainmask with grey and white matter segmentation, alongside pial and white matter surfaces, (E) Coronal SynthSR Raw image used for quality control, showing pial and white matter surfaces, (F) Coronal reconstruction of brainmask with grey and white matter segmentation, alongside pial and white matter surfaces

For quality control of the white matter surface, the raw SynthSR T1 image and WM mask were examined by both a PhD student and a radiologist with 15 years of experience, alongside the white matter surface references for the left and right hemispheres. Every slice of each view was then visually inspected to determine whether the white matter boundaries were accurately traced by the surface measurements, and any needed corrections were made. For the quality control of the pial surface, the raw SynthSR T1 and brainmask images were opened alongside the pial surface measurements. Each slice of each view was then checked to determine whether the surface accurately traced the edges of the brain and correctly measured the sulcal folds. This consists of ensuring the sulci aren’t skimmed over, and that non-grey matter tissue such as the dura are not included. Then, when any corrections were made, the process was re-run to ensure that the white matter and pial surface files accurately traced the newly defined edges of the white matter and pial surface. Minor corrections were needed for two of the scans, in one case in the right superior-parietal region, and another in the right middle frontal region. When major corrections were needed, this was typically accompanied by haemorrhaging which made corrections not possible, and so any major corrections were excluded. The white matter and pial surface tracing along the SynthSR raw image and brainmask can be seen in Fig. 1.

## Cortical lobe definitions and lobar thickness calculations

The different regions of the Desikan-Killiany atlas [19] which were separated for grouped measurement consisted of the frontal, parietal, temporal, and occipital lobes, alongside the cingulate gyrus and insula. The different regions of the FreeSurfer output which were included in each lobe can be see summarised in Supplementary Table 1.

The cortical volume and surface area for each lobe was calculated by aggregating the subregions. The Cortical thickness of each of the separated brain regions was calculated using Area-weighted means, which is commonly used in papers grouping DK atlas sub-regions together [26, 27]. In this method, the cortical thickness of each subregion is weighted based on the underlying surface area, allowing for an accurate calculation of the mean thickness of the lobes measured. This process can be seen in the following equation:

$$\:\mathrm{Lobe}{\mathrm{Thickness}}_{\mathrm{Weighted}}=\frac{{\sum\:}_{i=1}^{N}\left({\mathrm{Thickness}}_{i}\times\:{\mathrm{Area}}_{i}\right)}{{\sum\:}_{i=1}^{N}{\mathrm{Area}}_{i}}$$

### Statistical analysis

Statistical analyses were conducted using R software ver. 4.4.3 (R Foundation for Statistical Computing, Vienna, Austria) [28]. Participants were organised into individually matched sets, with each matched set comprising the participant with TBI at the initial scan, the same participant at the follow-up scan, and an individually age- and sex-matched neurotypical control participant. Each matched set was assigned a common ID, which was used as the repeated measures/blocking factor in the repeated measures ANOVA. The ANOVA investigated the effect of case group (Control, TBI initial and TBI follow-up) across the different cortical regions. Follow-up pairwise group comparisons were conducted using Wilcoxon Signed rank tests. Comparisons between the initial and follow-up scans were paired within the same participant, whereas comparisons between neurotypical control group and the TBI group at each time-point were based on individually age- and sex-matched control-TBI pairs. Non-parametric paired tests were used due to the small sample sizes. Effect sizes for the paired comparisons were calculated using paired-samples Cohen’s d, defined as the mean of the within-pair differences divided by the standard deviation of those differences. Cohen’s d was calculated separately from the Wilcoxon Signed-rank test and was interpreted as moderate (d = 0.50), or large (d > 0.80). The longitudinal differences in the individuals with TBI were then visually represented using the package “ggplot2” (ver. 4.0.3) for lobar regions, and “ggseg” (ver. 1.6.8) for the anatomical mapping of the DK atlas. Additional figure layout and annotation were performed using the cowplot (ver. 1.2.0) and grid (ver. 4.4.3) packages.

## Results

Each of the measured brain regions was investigated as described in the Methods section. Three main case groups for the analysis, Case Group 1 (TBI at the initial scan), Case Group 2 (TBI at the follow-up scan), and Case Group 3 (age- and sex-matched neurotypical control). Repeated-measures ANOVA were conducted to investigate the effect of case group on cortical measures, including interactions with brain region. Effect sizes were using η², with η² >0.16 indicating large effect sizes and η² = 0.06 indicating moderate effect sizes, as summarised in Table 1. Wilcoxon Signed Rank tests were conducted to compare the cortical measures across the three case groups. These tests can be seen summarised, with Table 2 showing Cortical Volume, Table 3 showing Cortical Thickness, and Supplementary Table 2 showing Cortical Surface Area. Descriptive statistics summarising the means and standard deviations for the neurotypical control group, and group with TBI at initial and follow-up scans can also be found in each relevant table.

**Table 1:** Repeated-measured ANOVA results for cortical measures across case groups

Measure	Approx. F	Num Df	Den Df	*p*-value	η²
Thickness (Case Group)	11.83	1.39	11.10	0.003*	0.253
Thickness (Case Group x Brain Region)	3.47	22	176	< 0.001*	0.076
Volume (Case Group)	1.54	1.17	11.69	0.244	0.051
Volume (Case Group x Brain Region)	2.10	22	176	0.004*	0.061
Surface Area (Case Group)	0.06	1.36	10.90	0.882	0.001
Surface Area (Case Group x Brain Region)	0.60	22	176	0.922	0.017

**Table 2:** Descriptive statistics and Wilcoxon tests for Cortical Volume Measurements (in mm^3^), Wilcoxon Signed-rank tests conducted for paired analyses between TBI 1 vs. TBI 2, and for control comparisons

Brain Region	Mean ± SD			Wilcoxon Signed Rank Test z (d)		
	Control	TBI Initial Scan	TBI Follow-up Scan	Control vs. TBI Initial	Control vs. TBI Follow-up	TBI Initial vs. TBI Followup
Left Frontal	65,433 ± 5735	68,962 ± 8791	64,384 ± 6878	1.17 (0.40)	-0.25 (-0.13)	1.89 (0.72)
Right Frontal	67,841 ± 10,010	71,448 ± 9354	66,720 ± 6318	0.87 (0.26)	-0.56 (-0.11)	1.89 (0.73)
Left Parietal	36,813 ± 4494	36,376 ± 3346	36,547 ± 4933	-0.76 (-0.08)	-0.36 (-0.04)	-0.25 (-0.08)
Right Parietal	36,624 ± 5654	37,426 ± 4859	35,963 ± 4241	0.15 (0.14)	-0.05 (-0.10)	1.78 (0.63)
Left Temporal	56,850 ± 6276	62,836 ± 9877	57,255 ± 4932	1.68 (0.62)	-0.05 (0.05)	2.09 (0.76)
Right Temporal	59,516 ± 8504	64,241 ± 6207	59,411 ± 5149	1.58 (0.62)	0.15 (-0.02)	2.50 (1.25)
Left Occipital	18,806 ± 1670	19,965 ± 1962	19,437 ± 2309	1.58 (0.57)	0.87 (0.26)	0.97 (0.34)
Right Occipital	22,868 ± 4002	25,099 ± 2136	24,173 ± 2394	1.48 (0.56)	1.38 (0.35)	1.68 (0.55)
Left Cingulate Gyrus	12,213 ± 1497	12,660 ± 1537	11,512 ± 1964	0.05 (0.16)	-0.76 (-0.24)	1.99 (0.83)
Right Cingulate Gyrus	14,794 ± 2727	15,051 ± 2489	13,291 ± 1539	-0.36 (0.07)	-1.38 (-0.42)	2.19 (0.88)
Left Insula	10,083 ± 1028	10,743 ± 870	10,793 ± 2088	1.27 (0.52)	0.97 (0.40)	0.97 (-0.04)
Right Insula	8890 ± 1022	9304 ± 1065	8989 ± 932	0.97 (0.33)	0.46 (0.10)	1.17 (0.44)

**Table 3:** Descriptive statistics and Wilcoxon tests for Cortical Thickness Measurements (in mm), Wilcoxon Signed-rank tests conducted for paired analyses between TBI 1 vs. TBI 2, and for control comparisons

Brain Region	Mean ± SD			Wilcoxon Signed Rank Test z (d)		
	Control	TBI Initial Scan	TBI Follow-up Scan	Control vs. TBI Initial	Control vs. TBI Follow-up	TBI Initial vs. TBI Followup
Left Frontal	2.55 ± 0.07	2.65 ± 0.16	2.51 ± 0.09	1.99 (0.78)	-1.07 (-0.45)	2.60 (1.38)
Right Frontal	2.64 ± 0.06	2.72 ± 0.19	2.69 ± 0.13	1.58 (0.48)	1.68 (0.55)	1.07 (0.14)
Left Parietal	2.33 ± 0.05	2.40 ± 0.1	2.32 ± 0.08	2.29 (0.92)	-0.25 (-0.08)	2.09 (0.92)
Right Parietal	2.40 ± 0.05	2.50 ± 0.13	2.44 ± 0.08	1.78 (0.79)	1.58 (0.47)	1.27 (0.53)
Left Temporal	2.69 ± 0.07	2.84 ± 0.15	2.73 ± 0.1	2.29 (0.90)	1.27 (0.39)	2.40 (0.89)
Right Temporal	2.79 ± 0.06	2.95 ± 0.19	2.85 ± 0.11	2.50 (0.85)	1.58 (0.60)	1.48 (0.50)
Left Occipital	2.26 ± 0.13	2.34 ± 0.12	2.24 ± 0.1	1.89 (0.75)	-0.36 (-0.17)	2.70 (1.35) *
Right Occipital	2.30 ± 0.09	2.49 ± 0.23	2.36 ± 0.1	2.50 (0.88)	1.58 (0.51)	1.58 (0.60)
Left Cingulate Gyrus	2.62 ± 0.05	2.74 ± 0.19	2.52 ± 0.15	0.97 (0.56)	-1.78 (-0.57)	2.60 (1.41)
Right Cingulate Gyrus	2.60 ± 0.06	2.77 ± 0.29	2.54 ± 0.15	1.58 (0.60)	-1.27 (-0.43)	2.70 (1.14) *
Left Insula	2.81 ± 0.05	3.03 ± 0.19	2.88 ± 0.08	2.70 (1.29) *	2.09 (0.84)	2.19 (0.85)
Right Insula	2.86 ± 0.06	3.09 ± 0.26	2.91 ± 0.09	2.09 (0.92)	1.68 (0.56)	2.09 (0.74)

*p<0.004 for significance after Bonferroni correction for multiple comparisons

## Repeated measures ANOVA

A repeated-measures ANOVA with brain region included as a within-subjects factor, revealed no significant main effect of case group on cortical surface area (F(1.36, 10.90) = 0.06, *p* = 0.882, η² = 0.001), and no significant interaction between case group and brain region (F(22, 176) = 0.60, *p* = 0.922, η² = 0.017), indicating an absence of both global and region-specific differences across groups.

In contrast, repeated-measures ANOVA for cortical volume revealed no significant main effect of case group (F(1.24, 9.92) = 2.83, *p* = 0.12, η² = 0.051), but a significant interaction of moderate size between case group and brain region (F(22, 176) = 2.10, *p* = 0.004, η² = 0.061), indicating that regional volume differences across brain regions varied across case groups.

For cortical thickness, repeated-measures ANOVA revealed a large significant main effect of case group (F(1.39, 11.10) = 11.83, *p* = 0.003, η² = 0.253), as well as a significant interaction of moderate size between case group and brain region (F(22, 176) = 3.47, *p* < 0.001, η² = 0.076), indicating both global and region-specific differences in thickness across case groups.

### Pairwise comparisons

To assess pairwise group differences, Wilcoxon signed rank tests were used, while applying a Bonferroni correction on the p-values to correct for multiple comparisons. Effect sizes were reported using paired Cohen’s d, with d > 0.80 indicating large effect sizes and d = 0.50 indicating a moderate effect size.

When investigating cortical surface area, there were no significant differences when comparing the paired scans of the individuals with TBI and when comparing the neurotypical control group with the group with TBI at both initial and follow-up scan. Effect sizes were small across regions as seen in Supplementary Table 2.

For cortical volume, Wilcoxon signed-rank analyses indicated significant group differences in several regions when comparing the initial and follow-up scans in individuals with TBI, as well as when comparing neurotypical controls with individuals with TBI at initial scan. However, these findings did not survive Bonferroni correction for multiple comparisons. Effect sizes ranged from small to moderate across regions, as presented in Table 2.

**Fig. 2:**
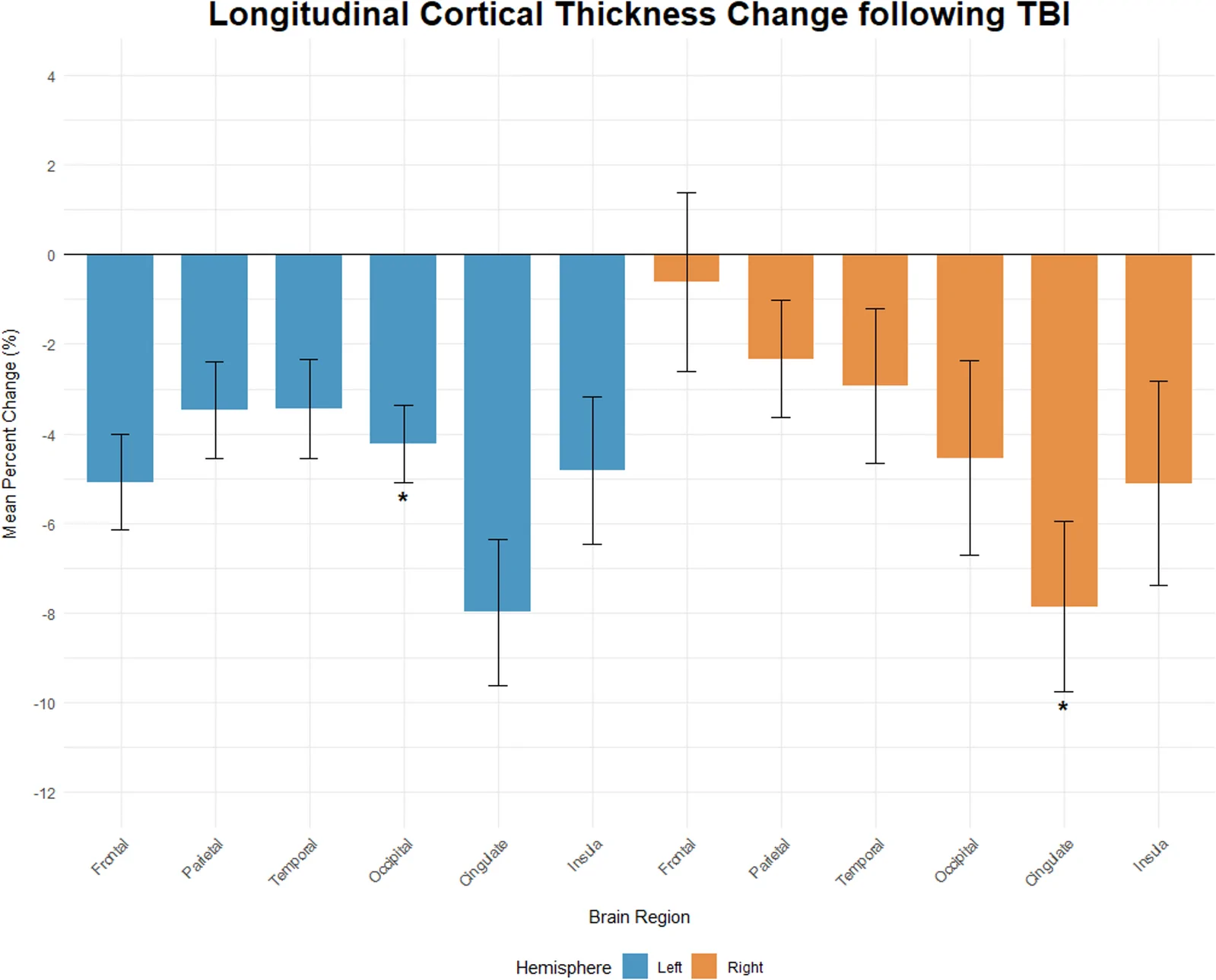
Bar plot showing longitudinal thickness changes across brain regions following TBI (*n* = 10)

**Fig. 3:**
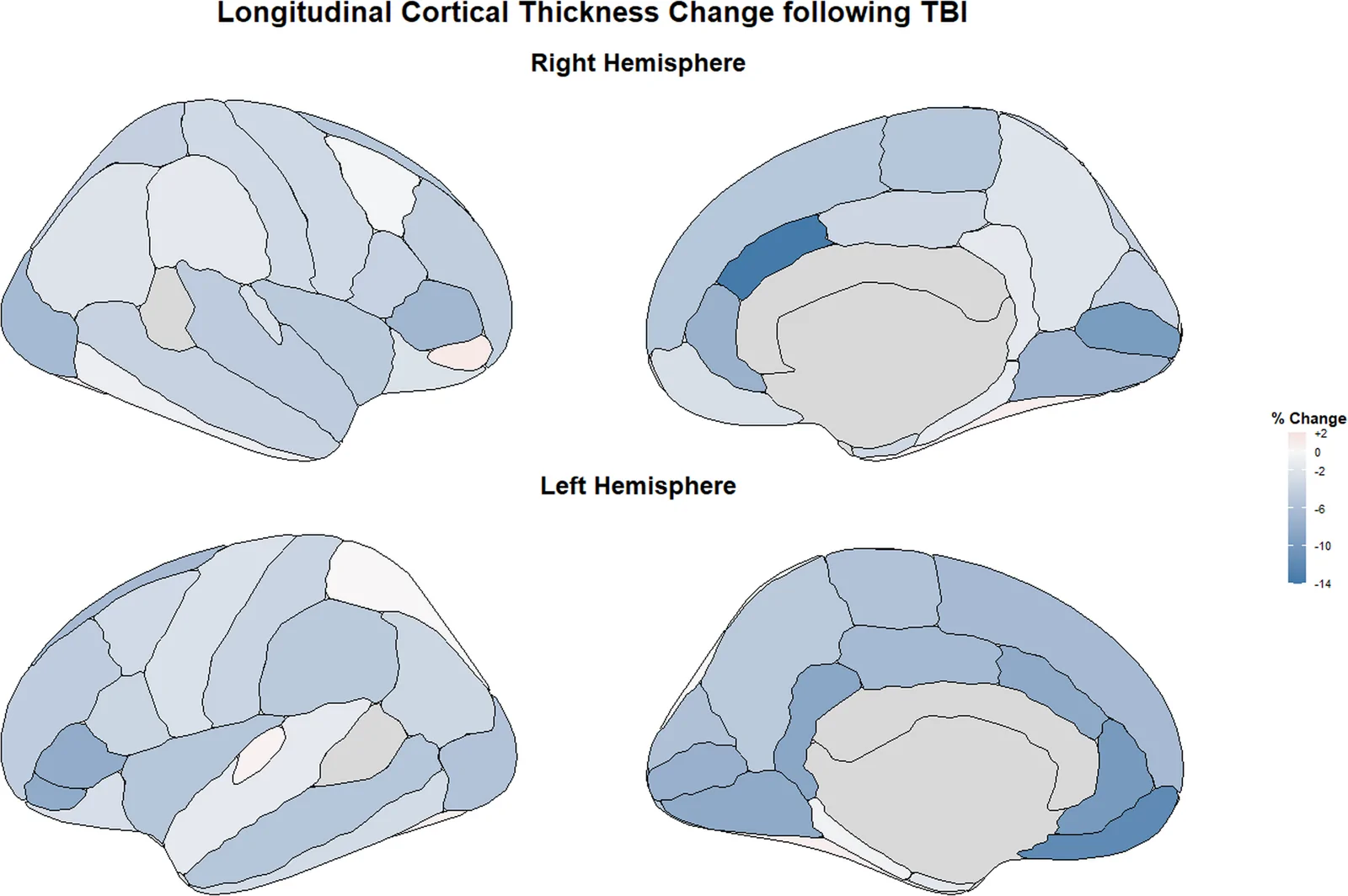
Mean longitudinal percentage change in cortical thickness between initial and follow-up MRI scans in the group with TBI (*n* = 10), visualised using the Desikan-Killiany atlas. Lateral and medial cortical surfaces are shown for both hemispheres. Colour intensity represents the magnitude of cortical thickness change from baseline to follow-up, with blue indicating cortical thinning and red indicating cortical thickening. Grey regions indicate unavailable or non-applicable data. Specifically, the bankssts, frontal pole, temporal pole, corpus callosum and medial wall are displayed in grey because these regions were not present in the exported FreeSurfer dataset and were therefore not included in the analysis. A numerically labelled version of this figure can be seen in Supplementary Fig. 2, with the labels in Supplementary Table 3

For cortical thickness, Wilcoxon signed-rank analyses demonstrated multiple significant region-specific differences when comparing controls with individuals with TBI at the initial scan, a single difference at follow-up, and several differences between the initial and follow-up scans in individuals with TBI. Effect sizes ranged from moderate to large across these comparisons. However, these findings were largely removed following Bonferroni correction, with only three comparisons remaining significant; Control vs. TBI initial in the left insula, and TBI initial vs. TBI follow-up in the left occipital cortex and right cingulate gyrus. The longitudinal changes seen across the lobes can be seen in Fig. 2, showing the mean percentage change of each grouped brain regions between initial and follow-up scans, and these changes are visualised in Fig. 3.

## Discussion

In this study, post-traumatic changes in cortical morphometry were measured using the “recon-all-clinical” pipeline on FreeSurfer, allowing for measurements of cortical volume, cortical thickness and cortical surface area. Due to the use of 4 mm MRI scans, the specific volume, thickness and surface area of the gyri and sulci in the DK atlas [19] were grouped into lobar regions consisting of the frontal, parietal, occipital and temporal lobes, alongside the cingulate gyrus and insula regions by using area-weighted means as shown in the methods section. This was also done to reduce the false discovery rate and type 1 errors, and to limit findings to specific lobes with clearer clinical implications for morphometric changes. Bonferroni corrections for multiple comparisons were applied to pairwise comparisons to further reduce the rate of type 1 errors.

Repeated-measures ANOVA demonstrated significant effects for cortical thickness, including both global and region-specific differences. In contrast, cortical volume showed no significant global effect, but demonstrated region-specific differences, while cortical surface area showed no significant global or region-specific differences across case groups. As hypothesised, there were longitudinal reductions in cortical morphometry, with pairwise comparisons confirmed several differences across various brain regions. However, after applying Bonferroni corrections for multiple comparisons, these changes were limited to cortical thickness, with no significant changes being seen for cortical volume or cortical surface area. Following analysis comparing the longitudinal cortical volume measurements across TBI scans, there were several differences in volume, however these differences did not survive the Bonferroni correction for multiple comparisons. These findings do not support previous research finding global cortical volume reductions following mTBI [7] and moderate-severe TBI [6]. However, these findings do support previous research by Ling et al., [11] which found no longitudinal reductions in cortical volume 2-weeks to 4-months post-TBI. However, when investigating this further by separating cortical volume into cortical surface area and cortical thickness, the cause of the volume reductions could be seen in more detail. For cortical surface area, this study found no significant differences in any regions between TBI time-points or control scans; this finding is somewhat consistent with prior research finding sparse increases and decreases across regions such the right lateral occipital gyrus orbitofrontal cortex and posterior regions of the brain [12, 13].

Cortical thickness appeared to be the morphometric feature impacted the most by TBI in this cohort, whereby longitudinal analysis of the individuals with TBI showed significant decreases in cortical thickness across multiple regions. However, following Bonferroni corrections, only the left occipital cortex and right cingulate gyrus remained significantly reduced. Prior studies have found significant longitudinal reductions in cortical thickness across various portions of the frontal lobe, including the left rostral middle frontal area, left caudal middle frontal, and left lateral orbitofrontal area in TBI groups [14]. Others have reported cortical thickness reductions in the fronto-temporal regions following mTBI [4], however these findings were not observed in the present study. Similarly, the current findings do not support prior reports of cortical thinning in the right hemispheric insula and the left middle temporal region, right parietal or temporal regions [15, 16]. The results of this study do somewhat support prior findings of cortical thickness reductions in the posterior cingulate region [12], however their study found longitudinal reductions in the precuneus regions, and increases in frontal thickness which were not observed here. The variance in findings may be driven by sample differences, however, may also be due to the separation of TBI severities in these studies [15, 16], compared to the present study’s inclusion of roughly equal mild and moderate-severe individuals with TBI due to sample restraints. In addition, the use of conservative statistical correction in the present study may have contributed to the reduced support of prior research. Overall, these findings indicate that cortical thickness changes following TBI may be more regionally specific, and less widespread than previously reported. However, these results should be interpreted with caution considering the limited sample size and statistical corrections applied.

When analysing control-TBI comparisons, individuals with TBI at both initial and follow-up scans showed no significant differences in cortical volume. These findings do not support previous reports of widespread volume reductions spread across the frontal, parietal and occipital lobes in some studies [8], and others which found reductions to portions of the cingulate gyrus, right insula, and bilateral frontal and temporal lobes [9]. In contrast, comparisons of cortical thickness between the neurotypical control and individuals with TBI at baseline revealed increases in cortical thickness across several regions. However, following Bonferroni corrections, only the left insula remained significant, and no significant differences remained when comparing the control with TBI follow-up. This does not support the hypothesis that cortical surface area, thickness and volume would be reduced in individuals with TBI at both time-points when compared with controls. These findings do not support previous research which found increases in cortical thickness of the left rostral middle frontal area longitudinally in individuals with TBI [14]. However, in their study they discuss that increases in regional cortical thickness may reflect inflammatory responses such as gliosis and swelling which may exhibit erroneous volume increases [14, 29]. A similar explanation is possible in the present study, whereby inflammatory processes such as gliosis or swelling may contribute to increased cortical thickness and volume in regions adjacent to the site of injury. However, as no direct biological or inflammatory measures were available, this interpretation remains speculative, and other mechanisms cannot be excluded. This may suggest that although all the cases were 4 mm axial T1 scans for consistency, further validation of the “recon-all-clinical” pipeline is warranted for detailed morphometric analysis of 4 mm TBI scans, as acute post-traumatic biological changes, including inflammatory processes, may influence cortical measurements and complicate comparisons between controls and individuals with TBI. The results of this study suggest that following TBI, changes to cortical volume may be due to changes in cortical thickness as opposed to cortical surface area; future studies should investigate this further by separating cortical volume into surface area and thickness to verify any relationships which may occur. Further validation studies using the “recon-all-clinical” pipeline should be conducted, with larger samples and include demographic and clinical information to investigate further what may specific patient features may increase the risk of cortical thinning following TBI.

Limitations of this study include the lack of separation of mild and moderate-severe TBI; this was not done as the sample sizes of these separated groups would be quite small and impact the power of the analysis. Another limitation with the retrospective study design is the variance in follow-up scan time; while 9 of the follow-ups were between 4 and 24 months, three extended to longer intervals. Although the TBI and control groups were age-matched at baseline, age-related cortical changes may still contribute to within-group variability in longitudinal measures. Visual inspection of the distribution of age and follow-up duration does not indicate a clear systematic relationship between these variables (see Supplementary Fig. 1). While one older participant had a relatively long follow-up period, this pattern is not consistently observed across the sample. Nevertheless, age and follow-up duration were not included as covariates in the statistical models due to the small sample size and should be acknowledged as potential sources of variability. If a follow-up scan of the controls within the timeframe could have been acquired, this would have improved the results and comparisons of this study as well. Due to ethics restrictions and the retrospective study design, we were unable to acquire clinical information from the patients besides GCS. Features such as loss of consciousness duration, TBI sub-types and localised injury location were not known to the researchers, and so may have contributed to the changes reported in this study.

This study investigated the use of the “recon-all-clinical” pipeline in 4 mm slice thickness scans; this study consisted of individuals with TBI at initial and follow-up scan, and age/sex matched controls with a single scan. This study found significant cortical thinning over time in the group with TBI in the left occipital cortex and right cingulate gyrus, while finding no changes in cortical surface area or volume in any region following multiple comparisons corrections. However, comparisons between the neurotypical control group and the group with TBI revealed increases in cortical thickness in the left insula; raising the possibility that acute post-traumatic biological processes, including inflammatory responses, may have contributed to the observed increases in cortical thickness. While the cortical segmentation produced using “recon-all-clinical” appeared internally consistent when scans were acquired at the same slice thickness, due to the complexity of cortical changes following TBI this pipeline requires additional research using a larger sample to verify the validity of short-term cortical morphometry following TBI. However, additional validation is required to determine the performance of this pipeline across different stages following TBI, particularly during the acute phase when post-traumatic biological changes may influence morphometric measurements. “recon-all-clinical” undoubtedly represents a breakthrough in neuroimaging measurement and will expand the available resources which can be measured, however future researchers using this pipeline to investigate individuals with TBI should interpret short-term cortical morphometry cautiously until further validation studies are available.

## Supplementary Information

Below is the link to the electronic supplementary material.

Supplementary Material 1 (PNG 736 KB)

Supplementary Material 2 (DOCX 192 KB)

## Data Availability

The data are available upon request.
