# Supplementary material for: MRI-based cortical morphometry in traumatic brain injury: a pilot longitudinal study
Source: Neuroradiology. 2026 Aug 3;68(9):2793–803. doi: 10.1007/s00234-026-04140-3 (PMC13680297; doi:10.1007/s00234-026-04140-3)
Supplement: Supplementary file 1 [file 234_2026_4140_MOESM2_ESM.docx]

Supplementary Figure 1. Scatterplot comparing age with follow-up duration in group with TBI

Supplementary Figure 2. Numerically labelled version of Figure 3 from the text, the associated numbers for this figure can be seen in Supplementary Table 3.

Supplementary Table 1. Gyri and Sulci included in each grouped cortical region

| Brain Region: | Included Areas |
| --- | --- |
| Frontal Lobe | Frontomarginal Gyrus and Sulcus, Gyrus Frontal Inferior Opercular Part, Gyrus Frontal Inferior Orbital Part, Gyrus Frontal Inferior Triangular Part, Gyrus Frontal Middle, Gyrus Frontal Superior, Gyrus Precentral, Sulcus Frontal Inferior, Sulcus Frontal Middle, Sulcus Frontal Superior, Sulcus Intermedius Primus (of Jensen), Gyrus Rectus, Gyrus Subcallosal, Gyrus Orbital, Gyrus and Sulcus Transverse Frontopolar, Sulcus Precentral Inferior Part, Sulcus Precentral Superior Part. |
| Parietal Lobe | Gyrus Parietal Inferior Angular, Gyrus Parietal Inferior Supramarginal, Gyrus Parietal Superior, Gyrus Postcentral, Sulcus Intraparietal and Parietal Transverse, Gyrus Precuneus, Sulcus Subparietal, Sulcus Postcentral. |
| Temporal Lobe | Gyrus Temporal Superior and Gyrus Transverse Temporalis, Gyrus Temporal Superior Lateral, Gyrus Temporal Superior Planum Polare, Gyrus Temporal Superior Planum Temporale, Gyrus Temporal Middle, Gyrus Temporal Inferior, Sulcus Temporal Inferior, Sulcus Temporal Superior, Sulcus Temporal Transverse, Pole Temporal, Gyrus Occipito-Temporal Lateral Fusiform, Gyrus Occipito-Temporal Medial Lingual, Gyrus Occipito-Temporal Medial Parahippocampal, Sulcus Occipito-Temporal Lateral, Sulcus Occipito-Temporal Medial and Lingual. |
| Occipital Lobe | Gyrus and Sulcus Occipital Inferior, Gyrus Cuneus, Gyrus Occipital Middle, Gyrus Occipital Superior, Sulcus Occipital Middle and Lunatus, Sulcus Occipital Superior and Transversal, Pole Occipital, Sulcus Occipital Anterior. |
| Cingulate Gyrus | Gyrus and Sulcus Cingulate Anterior, Gyrus and Sulcus Cingulate Mid-Anterior, Gyrus and Sulcus Cingulate Mid-Posterior, Gyrus Cingulate Post-Dorsal, Gyrus Cingulate Post-Ventral, Sulcus Cingulate Marginalis. |
| Insula | Gyrus Insulae Longus and Sulcus Centralis Insulae, Gyrus Insular Short, Sulcus Circularis Insulae Anterior, Sulcus Circularis Insulae Superior, Sulcus Circularis Insulae Inferior, Sulcus Suborbital. |

Supplementary Table 2. Descriptive statistics and Wilcoxon tests for Cortical Surface Area Measurements (in mm^2^), Wilcoxon Signed-rank tests conducted for paired analyses between TBI 1 vs TBI 2, and for control comparisons.

|  | Mean ± SD | | | Wilcoxon Signed Rank Test z (d) | | |
| --- | --- | --- | --- | --- | --- | --- |
| Brain Region | Control | TBI Initial Scan | TBI Follow-up Scan | Control vs TBI Initial | Control vs TBI Follow-up | TBI Initial vs TBI Followup |
| Left Frontal | 22446 ± 1970 | 22186 ± 2133 | 22372 ± 2286 | -0.25 (-0.13) | -0.15 (-0.02) | -0.36 (-0.08) |
| Right Frontal | 22014 ± 3267 | 21748 ± 2267 | 20747 ± 2232 | -0.46 (-0.08) | -0.87 (-0.32) | 1.38 (0.47) |
| Left Parietal | 14086 ± 1723 | 13319 ± 736 | 13907 ± 1624 | -1.78 (-0.53) | -0.66 (-0.07) | -1.17 (-0.44) |
| Right Parietal | 13325 ± 2035 | 12711 ± 968 | 12815 ± 1445 | -1.48 (-0.35) | -0.87 (-0.20) | -0.25 (-0.11) |
| Left Temporal | 18358 ± 2045 | 18470 ± 2084 | 17923 ± 1229 | -0.05 (0.06) | -0.46 (-0.17) | 0.87 (0.25) |
| Right Temporal | 18420 ± 2652 | 17917 ± 1740 | 17689 ± 2023 | -0.97 (-0.31) | -1.07 (-0.35) | 0.66 (0.12) |
| Left Occipital | 7818 ± 1064 | 7938 ± 1015 | 8112 ± 1064 | 0.36 (0.17) | 0.66 (0.34) | -0.97 (-0.25) |
| Right Occipital | 9244 ± 1760 | 9175 ± 1423 | 9397 ± 934 | -0.15 (-0.05) | 0.76 (0.11) | -0.25 (-0.25) |
| Left Cingulate Gyrus | 4564 ± 521 | 4530 ± 517 | 4456 ± 551 | -1.07 (-0.05) | -0.66 (-0.12) | 0.46 (0.15) |
| Right Cingulate Gyrus | 5483 ± 872 | 5199 ± 676 | 5000 ± 447 | -1.38 (-0.38) | -1.48 (-0.45) | 0.66 (0.29) |
| Left Insula | 3853 ± 406 | 3813 ± 334 | 3909 ± 432 | -0.46 (-0.10) | 0.15 (0.12) | -1.17 (-0.48) |
| Right Insula | 3280 ± 386 | 3160 ± 487 | 3202 ± 296 | -0.97 (-0.36) | -0.76 (-0.29) | -0.87 (-0.16) |

*p<.004 for significance, #p<.008 for trending significance after Bonferroni correction for multiple comparisons

Supplementary Table 3. Numerical associations for Supplementary Figure 2

| Number | Region |
| --- | --- |
| 1 | Bankssts |
| 2 | Caudal anterior cingulate |
| 3 | Caudal middle frontal |
| 4 | Cuneus |
| 5 | Entorhinal |
| 6 | Frontal pole |
| 7 | Fusiform |
| 8 | Inferior parietal |
| 9 | Inferior temporal |
| 10 | Insula |
| 11 | Isthmus cingulate |
| 12 | Lateral occipital |
| 13 | Lateral orbitofrontal |
| 14 | Lingual |
| 15 | Medial orbitofrontal |
| 16 | Middle temporal |
| 17 | Paracentral |
| 18 | Parahippocampal |
| 19 | Pars opercularis |
| 20 | Pars orbitalis |
| 21 | Pars triangularis |
| 22 | Pericalcarine |
| 23 | Postcentral |
| 24 | Posterior cingulate |
| 25 | Precentral |
| 26 | Precuneus |
| 27 | Rostral anterior cingulate |
| 28 | Rostral middle frontal |
| 29 | Superior frontal |
| 30 | Superior parietal |
| 31 | Superior temporal |
| 32 | Supramarginal |
| 33 | Temporal pole |
| 34 | Transverse temporal |
| 35 | Corpus callosum |
| 36 | Medial wall |
